# Efficacy of Enhanced Recovery After Surgery Program for Patients Undergoing Lobectomy for Lung Cancer: A Scoping Review and Single-Center Initial Result in Vietnam

**DOI:** 10.7759/cureus.44084

**Published:** 2023-08-25

**Authors:** Ho Tat Bang, Tran Thi Anh Thu, Vo Hieu Nghia, Tran Khanh Huyen, Tran Thanh Vy, Nguyen Van Tap

**Affiliations:** 1 Thoracic and Vascular Department, University Medical Center Ho Chi Minh City, University of Medicine and Pharmacy at Ho Chi Minh City, Ho Chi Minh City, VNM; 2 Department of Health Organization and Management, Faculty of Public Health, University of Medicine and Pharmacy at Ho Chi Minh City, Ho Chi Minh City, VNM; 3 Faculty of Public Health, University of Medicine and Pharmacy at Ho Chi Minh City, Ho Chi Minh City, VNM; 4 Department of Health Communication, Medical Center of Binh Thanh District, Ho Chi Minh City, VNM; 5 Department of Planning, Dong Thap Center for Disease Control and Prevention, Cao Lanh City, VNM; 6 Department of Health Communication, Medical Center of Tan Phu District, Ho Chi Minh City, VNM; 7 Faculty of Medicine, University of Medicine and Pharmacy at Ho Chi Minh City, Ho Chi Minh City, VNM; 8 Faculty of Medical Management, Nguyen Tat Thanh University, Ho Chi Minh City, VNM

**Keywords:** efficacy, length of stay, eras, lung cancer, lobectomy

## Abstract

Surgery for lung cancer can be invasive and the recovery process is often slow with many complications. To address this, the enhanced recovery after surgery (ERAS) program aims to minimize adverse clinical events for surgical patients. This is achieved through a multimodal perioperative care protocol that aims to preserve preoperative organ function and reduce postoperative complications. Initially applied to gastrointestinal surgery, this model has now been expanded to other major surgeries, including lung surgery. Through a review of seven retrospective and prospective cohort observational studies, we have examined the effects of the ERAS program on patients undergoing lobectomy for lung cancer treatment. Our analysis focused on outcomes such as length of stay, re-operation rate, re-admission rate, postoperative mortality, and costs, providing valuable insights into the real clinical practice setting. We also report on some initial results when applying ERAS at University Medical Center Ho Chi Minh City.

## Introduction and background

According to the 2020 statistics of GLOBOCAN, lung cancer ranked second in total cases with 11.4% and first in number of deaths with 1.8 million cases [1]. Currently, in the multimodal strategy for lung cancer treatment, surgery is one of the essential treatment methods for early-stage tumors. Lobectomy is the standard technique that is performed using either open surgery or video-assisted thoracoscopic surgery (VATS). However, evidence suggests that both surgical approaches are associated with some serious complications after surgery [2-4].

In order to minimize adverse clinical events for surgical patients, the model of enhanced recovery after surgery (ERAS) was developed by Kehlet H in 1990 [5]. ERAS is a series of evidence-based, multimodal, and comprehensive interventions before, during, and after surgery for patients undergoing major surgery. This is designed to achieve early recovery by maintaining preoperative organ function and reducing postoperative complications. This program helps patients reduce the experience of prolonged fasting, pain, vomiting, and nausea after surgery compared to traditional care. ERAS was initially applied to gastrointestinal surgery, and this model has now been expanded to other major surgeries. It applies to patients with lobectomy in 2012-2015 [6]. As of 2019, the ERAS program has been further developed and recommended by the Enhanced Recovery After Surgery Society and the European Society of Thoracic Surgeons [7].

Many studies have evaluated the role of ERAS in patients with lobectomy and have shown positive results in reducing hospital stays, costs, and complications after surgery [8]. However, a number of other results have shown ambiguous effects of the ERAS program on lung surgery patients, and safety has not been verified [9].

In this study, we performed a review based on seven retrospective and prospective cohort observational studies, which contributed to reflecting and providing additional results in a real clinical practice setting on the effects of the ERAS program in patients undergoing lobectomy for lung cancer treatment, with outcomes of interest being the length of stay, reoperation rates, readmission rates, postoperative mortality, and costs. Key aspects of regimens to enhance postoperative recovery have been published consisting of elements divided into three phases, including the preoperative, intraoperative, and postoperative phases [10]. We also report on some initial results when applying ERAS at University Medical Center Ho Chi Minh City.

## Review

Search strategy

**Figure 1:**
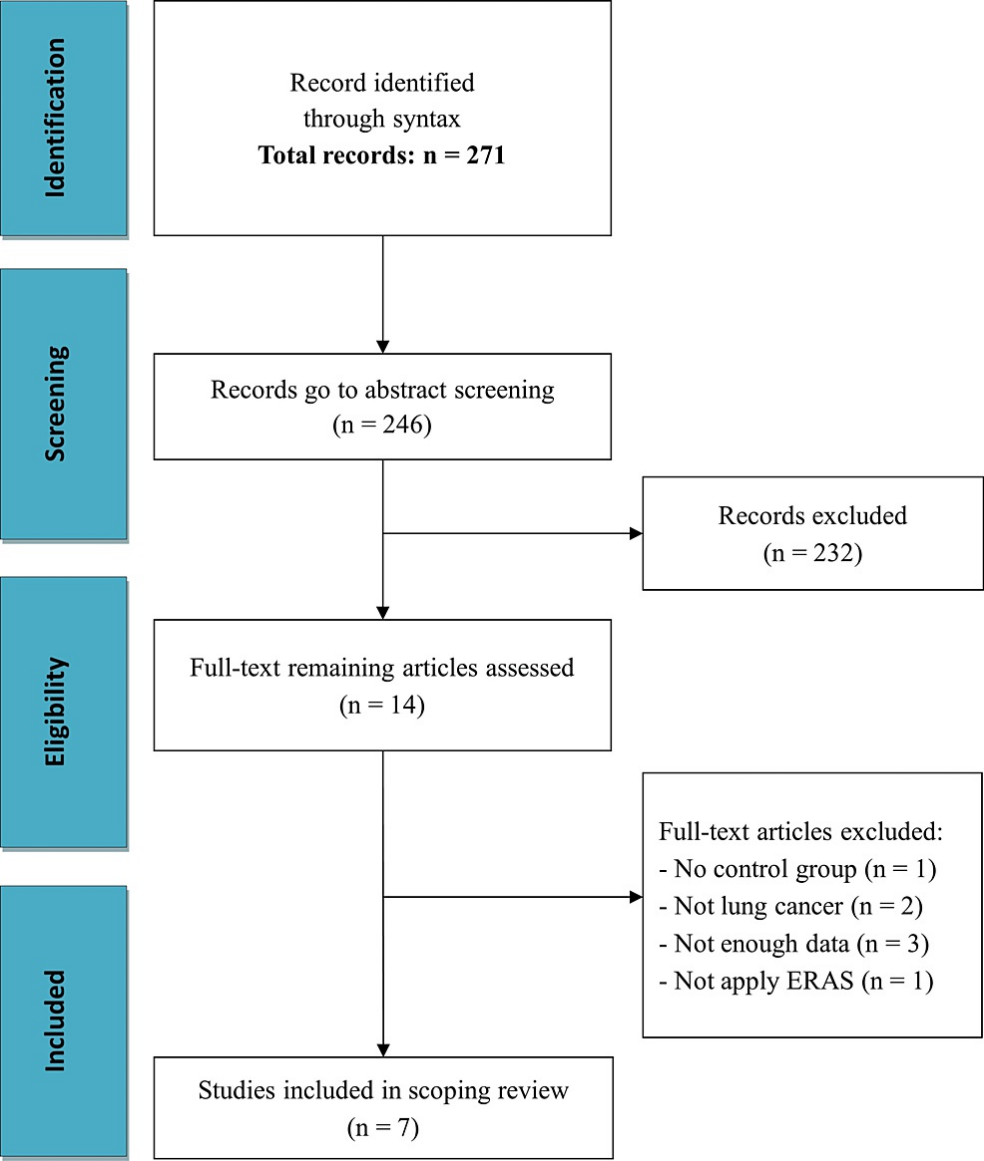
PRISMA flow diagram shows the study search and selection process PRISMA: preferred reporting items for systematic reviews and meta-analyses

The recommendations on preferred reporting items for systematic reviews and meta-analyses (PRISMA) were followed [11]. The PRISMA flow diagram, which presents the study search and selection process is shown in Figure 1. We searched papers on the PubMed database. The syntax was ("Pneumonectomy"[Mesh] OR lobectomy OR pneumonectomy OR Ablation lung OR Lung ablation) OR (Pulmonary Neoplasms OR Neoplasms, Lung OR Lung Neoplasm OR Neoplasm, Lung OR Neoplasms, Pulmonary OR Neoplasm, Pulmonary OR Pulmonary Neoplasm OR Lung Cancer OR Cancer, Lung OR Cancers, Lung OR Lung Cancers OR Pulmonary Cancer OR Cancer, Pulmonary OR Cancers, Pulmonary OR Pulmonary Cancers OR Cancer of the Lung OR Cancer of Lung OR "Lung Neoplasms"[Mesh]) AND (Restoration[All Fields] OR "Rehabilitation"[Mesh] OR "rehabilitation" [Subheading] OR Enhanced Postsurgical Recovery OR Postsurgical Recoveries, Enhanced OR Postsurgical Recovery, Enhanced OR Recovery, Enhanced Postsurgical OR "Enhanced Recovery After Surgery"[Mesh] OR enhanced OR Fast-track OR intensive OR Intensity Accelerated OR Recovery Rehabilitation OR Restoration OR Reinstatement) AND ("Length of Stay"[Mesh]); filters applied: English, from July 31, 2013 to July 31, 2023.

Selection criteria

We searched for studies that were designed as randomized controlled trials (RCTs) or cohorts with control groups. Articles without full text would be excluded. The results had to include one of the endpoints. Articles were manually screened through titles and abstracts. After discussion, two independent review authors decided to agree on articles that matched the identified inclusion criteria before inclusion in the scoping review. When a disagreement occurred, the final decision was made after a discussion with the supervisor.

Data extraction

Two independent reviewers extracted articles that met the inclusion criteria, and disagreements were resolved by discussion, and if they could not reach an agreement, the supervisor was consulted.

Data were extracted from articles, including author, year of publication, country of origin, and total number of study participants. We also collected information regarding the participants, including age, number of ERAS factors applied, and surgical method. Outcomes of interest included hospital days, re-operative rates, re-admission rates, postoperative mortality, and total costs.

Study endpoints

The primary outcome was the length of stay. The secondary outcomes were the re-operative rate, re-admission rate, postoperative mortality, and total cost. In our systematic review, the type of intervention we were interested in was the adoption of the ERAS program, regardless of whether the patient received open surgery or VATS. Two groups of patients were compared: the first group was the patients applying for the ERAS program, and the second group was the patients not applying for the ERAS program.

Length of stay is the time from the time the patient is operated on until the first discharge from the hospital. Re-operation is an event that recognizes the patient's need to continue surgery after being treated with previous surgical methods during a follow-up period. Re-admission is an event where a patient needs to be admitted to the hospital for treatment or to monitor their health condition after receiving previous surgical treatment during a follow-up period. Postoperative mortality is the event that records a patient's death after receiving surgical treatment during a follow-up period. Total costs are the actual costs associated with the provision of health care services by a patient, including costs for procedures, therapies, procedures, and medications during a hospital stay.

Search results

The total number of potential articles collected from PubMed was 271. Of these, 25 articles were removed after screening titles. After sifting articles through reading abstracts, 14 articles went to full-text review, and seven studies were eligible for inclusion in the scoping review. The detailed selection process is shown in the PRISMA flow diagram (Figure 1).

The characteristics of the studies and patients are detailed in Table 1. The main outcomes of the study included: length of hospital stay (7/7), re-admission (6/7), re-operation (2/7), mortality (5/7), and total cost (3/7).

**Table 1:** Details of the studies included in the review NSCLC: non-small cell lung cancer; VATS: video-assisted thoracoscopic surgery; N/I: no information; ERAS: enhanced recovery after surgery a: median (interquartile); b: median (range); c: median (interquartile range)

Author (year)	Country	Study population	Surgical method	Follow-up time after surgery	Intervention ERAS	Cases ERAS/ control	Age ERAS/Control	Main conclusion
Michel Gonzalez [2] (2018)	Switzerland	Patient 18 years old with a malignant lung tumor	VATS	30 days	16 elements	50 / 50	64 (44-87)^b^ / 68 (51-81)^b^	ERAS for VATS lung resection is cost-effective and is associated with a lower complication rate and a shorter length of stay
Céline Forster [4] (2021)	Switzerland	Patients 18 years of age with non-small cell lung cancer, regardless of stage	VATS	30 days	16 elements	140 / 167	67 (59-72)^a^ / 67 (60-74)^a^	ERAS for VATS lobectomies in NSCLC decreased the length of stay, and cardiopulmonary complication rate without affecting the re-admission rate
Chunmei Wang [3](2021)	China	Patient 18 years old with lung cancer	Open surgery VATS	N/I	14 elements	691 / 1058	61 (56-67)^a^ / 61 (53-68)^a^	ERAS for lung surgery shortened length of stay, lower in-hospital costs, and reduced pulmonary complications rate
Robert M. Van Haren [12] (2018)	USA	Primary lung cancer patient	Open surgery VATS	30 days	12 elements	342 / 1615	66 (13)^c^ / 65 (15)^c^	ERAS for lung surgery decreased the length of stay and cardiopulmonary complication rate after thoracotomy but not after VATS. ERAS safety was illustrated by low rates of adverse events without effect on hospital re-admission or perioperative deaths
Satoshi Shiono [13] (2019)	Japan	Elderly patient with lung malignancy	Open surgery	90 days	10 elements	130 / 405	70 (65-77)^a^ / 70 (63-77)^a^	ERAS for lung open surgery shortened the length of stay in elderly patients and did not increase re-admission rates
Greg J. Haro [14] (2021)	USA	Patients with primary or metastatic lung cancer	Open surgery VATS	30 days	15 elements	126 / 169	67 (59-72)^a^ / 67 (59-73)^a^	ERAS for elective lung resection reduced length of stay, morbidity, opioid use, and direct costs without changing the re-admission rate
Yahya Alwatari [15] (2021)	USA	Lung cancer patient	Open surgery	N/I	N/I	4080 / 2388	66.9 ± 9.3 / 67.2 ± 10.3	Patients in the ERAS group were less likely to experience unintended intubation, wound infection, and sepsis. Mortality rates were also significantly lower than in the pre-ERAS groups

All studies performed cohort study design. There were three studies conducted in the Americas (USA), two studies in Europe, and two studies in Asia. The study population consisted of patients diagnosed with primary lung cancer. The median age of patients in all studies was 61 years and older. In particular, there is one study conducted on elderly subjects in Japan, with the median age in both groups being 70 years old.

Postoperative follow-up time was 30 days in four studies, 90-day follow-up in one study, and two studies that did not report follow-up duration. Two studies used VATS as a surgical approach, two used open surgery, and three used both. Six studies report the components of the ERAS program applied. The selected intervention consists of 10 to 16 elements.

ERAS effectiveness

In terms of length of stay the results in Table 2 help to compare the median length of hospital stay between the ERAS and control groups. The reductions ranged from 1 to 3 days. A statistically significant difference in the length of hospital stay between the comprehensive ERAS program and the control group was reported in all studies. In the ERAS group, the median hospital stay ranged from 3 to 5 days. For the control group, the median hospital stay ranged from 4 to 7 days. In terms of total costs (Table 2) three studies all recorded a statistically significant difference between the group applying for the ERAS program and the control group.

**Table 2:** Impact of ERAS on length of stay and total costs ERAS: enhanced recovery after surgery a: median (quartile range); b: median (interquartile range) Transitional period: can be considered as a trial period for the application of ERAS

Study	Length of stay				Total costs		
	ERAS group	Transitional period	Control group (Non-ERAS)	p-value	ERAS group	Control group (Non-ERAS)	p-value
Michel Gonzalez [2] (2018)	4 (1-16)^b^	-	7 (2-21)^b^	<0.001	15.945 (€) (15.094–17.546)^b^	20.360 (€) (19.123–22.935)^b^	<0.0001
Céline Forster [4] (2021)	5 (4-10)^a^	-	7 (5-12)^a^	0.04	^-^	^-^	^-^
Chunmei Wang [3](2021)	4 (2-6)^a^	-	6 (4-9)^a^	<0.001	46047.7 ¥ (39068.7-52733.8)^a^	47583 ¥ (43761.6-51839.6)^a^	<0.0001
Robert M. Van Haren [12] (2018)	4 (3)^a^	4 (3)^a^	5 (3)^a^	<0.001	-	-	-
Satoshi Shiono [13] (2019)	3 (3-83)^b^	-	4 (4-18)^b^	< 0.001	-	-	-
Greg J. Haro [14] (2021)	3.1	-	4.5	< 0.01	19.100$	23.100$	< 0.01
Yahya Alwatari [15] (2021)	6.6 ± 4.7	7.1 ± 5.1	8.1 ± 6.4	< 0.01	-	-	-

The rate of re-admission (Table 3) between the two groups decreased from 1.8% to 6% in the three studies that reported this indicator. However, the studies have not shown a statistically significant difference between the groups of patients applying the ERAS program compared with the control group. Re-operation (Table 3) was reported in two studies with a decrease of 0.2% in one study and 0.2% increase in this rate in one study after the application of the ERAS program. Similar to the rate of re-admission, this difference was not statistically significant. Mortality (Table 3) was the event of interest in five studies. The results of one study showed a reduction in mortality of 13.2% in the group applying the 15-factor ERAS program compared to the control group, with statistical significance p = 0.02.

**Table 3:** Impact of ERAS on the re-admission, re-operation, and postoperative mortality rate ERAS: enhanced recovery after surgery Transitional period: can be considered as a trial period for the application of ERAS

Study	Re-admission				Re-operation				Mortality			
	ERAS group	Transitional period	Control group (Non-ERAS)	p-value	ERAS group	Transitional period	Control group (Non-ERAS)	p-value	ERAS group	Transitional period	Control group (Non-ERAS)	p-value
Michel Gonzalez [2] (2018)	1 (2%)	-	1 (2%)	1	-	-	-	-	0	-	0	-
Céline Forster [4] (2021)	5 (3.6%)	-	9 (5.4%)	0.45	-	-	-	-	-	-	-	-
Chunmei Wang [3](2021)	-	-	-	-	-	-	-	-	4 (0.6%)	-	6 (0.6%)	1
Robert M. Van Haren [12] (2018)	3.80%	3.00%	3.30%	0.772	1.50%	2.40%	1.70%	0.432	0.80%	0.50%	0.60%	0.417
Satoshi Shiono [13] (2019)	4 (0.8%)	-	27 (6.7%)	0.304	-	-	-	-	0	-	30 days: 1 case (0.2%) 90 days: 5 cases (1.2%)	0.999
Greg J. Haro [14] (2021)	7.50%	-	5.10%	0.35	-	-	-	-	22.40%	-	36.00%	0.02
Yahya Alwatari [15] (2021)	340 (7.6%)	311 (13.6%)	-	0.69	231 (5.7%)	176 (4.8%)	125 (5.5%)	0.23	-	-	-	-

Discussion

Effectiveness of ERAS in Lung Lobectomy for Lung Cancer Surgery

Since the ERAS program was introduced and applied to patients undergoing major surgery, its effectiveness in reducing hospital stay, reducing complication rates, and improving patient experience has been reported in many studies in many different types of surgery [7]. From the first milestones, the ERAS program has grown stronger, the ERAS Society being established with the mission: "Develop perioperative care and to improve recovery through research, education, audit and implementation of evidence-based practice" [7]. Thanks to guidance from the association and experts, global health systems have begun to apply and report the results of the ERAS model. ERAS was initially applied to gastrointestinal surgery and gradually applied to other surgeries. In the field of lung surgery, guidelines have also been published by the ERAS Society in 2019 [7]. In this study, we only focused on analyzing the effects of ERAS on postoperative recovery in patients with lung lobectomy in the treatment of lung cancer.

There have been many studies on the effectiveness of ERAS worldwide. However, the effectiveness reported specifically in the group of patients with lobectomy is limited in the number of studies. In all seven studies, we found that applying the ERAS model had an impact on reducing the postoperative length of hospital stay for patients after lung cancer surgery compared to the non-ERAS group. The study by Gonzalez et al. is the earliest (2018) with a report on ERAS for patients undergoing VATS lobectomy for the treatment of primary and metastatic lung masses [2]. This study was conducted before the 2019 ERAS Society's Guideline was published [7]. However, we found that the ERAS factors (16 factors) in this study were quite similar to the standards that the ERAS Society's Guideline proposed. The compliance rate of the constituent elements of ERAS is also quite high; 11/16 elements achieve a compliance level greater than 80%. The effectiveness of the ERAS program in reducing hospital stays was also reported to be quite significant, averaging four days in compared with seven days in the control group. The limitation of this study was not an RCT-designed study, and the sample size was small.

The remaining studies also showed an effective reduction in hospital stay when applying ERAS. The ERAS model is effective because it includes many pre-, intra-, and postoperative procedures that are well coordinated among hospital specialties. Patients are also directly involved in the treatment process by following the guidelines of the clinician. The patient's condition before surgery is also optimized, and pain relief methods and patient experience are more concern. All of these contribute to effectively reducing the length of hospital stay, thereby saving costs and medical resources. Implementing an ERAS program in a health facility requires the synchronous cooperation of many health management and quality management activities. In order for hospitals to effectively implement the ERAS model, their management systems must ensure that staff complies with procedures that are scientifically designed and optimized. Additionally, the facilities must be equipped to support the successful application of the ERAS model.

Regarding the effectiveness of reducing the rate of re-hospitalization, rate of re-surgery, and mortality, further studies with larger samples are needed in order to clarify. Research by five authors, Gonzalez et al. (2018) [2], Forster et al. (2021) [4], Wang et al. (2021) [3], Robert et al. (2018) [12], and Satoshi et al. (2019) [13] did not find a statistically significant difference in re-admission, re-operation, and mortality rate between the ERAS and non-ERAS groups. Only a study by author Haro et al. (2021) showed a decrease in morbidity from 32% in the non-ERAS to 20% in the ERAS group. In addition, the rate of minor morbidity decreased significantly in the ERAS group (33% in non-ERAS and 19% in ERAS) [14]. More studies with larger sample sizes and randomized controlled clinical designs are needed to further clarify the effectiveness of ERAS in lobectomy in terms of reducing re-admission rates, re-operation rates, and mortality rates. In terms of cost, three studies reported by Gonzalez et al. (2018) [2], Wang et al. (2021) [3], and Haro et al. (2021) [14] all showed a statistically significant cost reduction between the ERAS and non-ERAS groups.

Single-Center Initial Results in Vietnam

**Figure 2:**
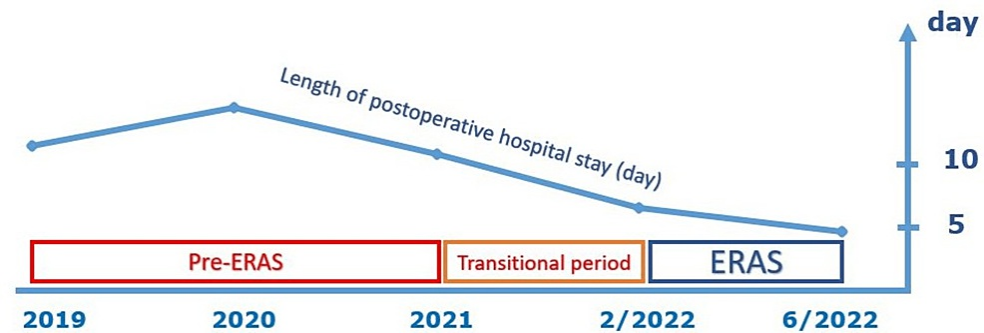
Length of hospital stay after (day) lobectomy in the treatment of lung cancer over the years at the University Medical Center Ho Chi Minh City (initial results)

At University Medical Center Ho Chi Minh City, in 2021, we started to develop ERAS procedures for a total of 14 types of surgery, including lobectomy for cancer treatment. In the transitional period, we established a network of ERAS members, prepared necessary facilities for ERAS (warmer, maltodextrin), developed standard operating procedures, checklists, patient information leaflets, and an electric medical records system. The initial survey of patients diagnosed with primary lung cancer participating in the ERAS program showed that hospital stay has gradually decreased compared to before the application (Figure 2). In the future, we will continue to improve the related processes and conduct studies to evaluate the effectiveness of the ERAS program more comprehensively. We will report the results in more detail in subsequent studies.

## Conclusions

A review of seven studies showed that the application of the ERAS program in lobectomy for cancer can help shorten the length of hospital stay after surgery. The other outcomes, including mortality, re-admissions, and re-operation, need to be studied more in the future. Initial results of ERAS applied at the University Medical Center Ho Chi Minh City also show that ERAS can shorten the postoperative length of hospital stay. The application of ERAS is completely feasible and has a high potential to reduce the overload of hospital beds.

## References

[1] Sung H, Ferlay J, Siegel RL, Laversanne M, Soerjomataram I, Jemal A, Bray F (2021). Global cancer statistics 2020: GLOBOCAN estimates of incidence and mortality worldwide for 36 cancers in 185 countries. CA Cancer J Clin.

[2] Gonzalez M, Abdelnour-Berchtold E, Perentes JY (2018). An enhanced recovery after surgery program for video-assisted thoracoscopic surgery anatomical lung resections is cost-effective. J Thorac Dis.

[3] Wang C, Lai Y, Li P, Su J, Che G (2021). Influence of enhanced recovery after surgery (ERAS) on patients receiving lung resection: a retrospective study of 1749 cases. BMC Surg.

[4] Forster C, Doucet V, Perentes JY (2021). Impact of an enhanced recovery after surgery pathway on thoracoscopic lobectomy outcomes in non-small cell lung cancer patients: a propensity score-matched study. Transl Lung Cancer Res.

[5] Kehlet H (1997). Multimodal approach to control postoperative pathophysiology and rehabilitation. Br J Anaesth.

[6] Steenhagen E (2016). Enhanced recovery after surgery: it’s time to change practice!. Nutr Clin Pract.

[7] Batchelor TJ, Rasburn NJ, Abdelnour-Berchtold E (2019). Guidelines for enhanced recovery after lung surgery: recommendations of the Enhanced Recovery After Surgery (ERAS®) Society and the European Society of Thoracic Surgeons (ESTS). Eur J Cardiothorac Surg.

[8] Nicholson A, Lowe MC, Parker J, Lewis SR, Alderson P, Smith AF (2014). Systematic review and meta-analysis of enhanced recovery programmes in surgical patients. Br J Surg.

[9] Huang L, Kehlet H, Petersen RH (2022). Reasons for staying in hospital after video-assisted thoracoscopic surgery lobectomy. BJS Open.

[10] Mazza F, Venturino M, Turello D (2020). Enhanced recovery after surgery: adherence and outcomes in elderly patients undergoing VATS lobectomy. Gen Thorac Cardiovasc Surg.

[11] Page MJ, McKenzie JE, Bossuyt PM (2021). The PRISMA 2020 statement: an updated guideline for reporting systematic reviews. Int J Surg.

[12] Van Haren RM, Mehran RJ, Mena GE (2018). Enhanced recovery decreases pulmonary and cardiac complications after thoracotomy for lung cancer. Ann Thorac Surg.

[13] Shiono S, Endo M, Suzuki K, Hayasaka K (2019). Impact of enhanced recovery after surgery on outcomes of elderly patients undergoing open thoracic surgery. Gen Thorac Cardiovasc Surg.

[14] Haro GJ, Sheu B, Marcus SG, Sarin A, Campbell L, Jablons DM, Kratz JR (2021). Perioperative lung resection outcomes after implementation of a multidisciplinary, evidence-based thoracic eras program. Ann Surg.

[15] Alwatari Y, Scheese D, Rustom S, Sevdalis AE, Ayalew D, Julliard W, Shah RD (2022). Trends in open lobectomy outcomes for lung cancer over the last 15 years: national cohort. Gen Thorac Cardiovasc Surg.

